# Weight change across adulthood in relation to the risk of depression

**DOI:** 10.3389/fpsyg.2023.1108093

**Published:** 2023-08-09

**Authors:** Tao Wang, Bingqin Dai, Huanchen Shi, Huawei Li, Kexin Fan, Dongfeng Zhang, Yunping Zhou

**Affiliations:** ^1^School of Health and Life Sciences, University of Health and Rehabilitation Sciences, Qingdao, China; ^2^Qingdao Hospital, University of Health and Rehabilitation Sciences (Qingdao Municipal Hospital), Qingdao, China; ^3^Shandong Provincial Center for Disease Control and Prevention, Jinan, China; ^4^School of Basic Medicine, Qingdao University, Qingdao, China; ^5^School of Nursing, Qingdao University, Qingdao, China; ^6^School of Public Health, Qingdao University, Qingdao, China

**Keywords:** obesity, weight change, NHANES, sex disparity, depression

## Abstract

**Background:**

Studies examining weight change patterns and depression are scarce and report inconsistent findings. This study—aimed to elucidate the association between weight change patterns and the risk of depression in a large, representative sample of US adults.

**Methods:**

Data from the National Health and Nutrition Examination Survey (NHANES) 2005–2018 was analyzed. Five weight change groups were categorized: stable normal, weight loss, weight gain, maximum overweight, and stable obesity. Depression was ascertained using the validated Patient Health Questionnaire (PHQ-9) and depression was defined as PHQ score ≥ 10.

**Results:**

A total of 17,556 participants were included. Compared with participants who maintained normal weight, stable obesity participants had increased risks of depression across adulthood from age 25 years to 10 years before the survey (OR = 1.61, 95% CI =1.23 to 2.11), in the 10 years period before the survey (OR = 2.15, 95% CI =1.71 to 2.70), and from age 25 years to survey (OR = 1.88, 95% CI =1.44 to 2.44). Weight gain was associated with an increased risk of depression from age 25 years to 10 years before the survey (OR = 1.71, 95% CI = 1.41 to 2.04), in the 10 years period before the survey (OR = 1.73, 95% CI = 1.35 to 2.21), and for the period from age 25 years to survey (OR = 1.83, 95% CI = 1.49 to 2.24). In the stratified analyses, we found statistically significant interactions with sex.

**Conclusion:**

Our study suggested that stable obesity and weight gain across adulthood were associated with increased risks of depression.

## 1. Introduction

According to the World Health Organization, more than 300 million people suffer from depression worldwide (Friedrich, 2017). Depression is a severe mental illness characterized by various psychological and physical symptoms, such as sadness for no reason, feelings of guilt, poor concentration, and sleep disorders (Ba et al., 2021). Depression has become one of the leading causes of disability and death (Chesney et al., 2014). Emerging evidence indicated that the pathophysiology of depression included a combination of genetic and/or biological factors such as obesity and the associated metabolic disorders.

Obesity is a growing health concern in many countries around the world and has been implicated in the development of depression. Previous studies have recognized that body mass index (BMI) was closely related to the risk of depression (Luppino et al., 2010; Pan et al., 2012; Anguita-Ruiz et al., 2022). Epidemiology studies indicated that high BMI in adulthood had been associated with an increased risk of depression. However, most previous research only included a single BMI measurement, ignoring the dynamic characteristics of body weight over time. Therefore, more research is needed to evaluate the long-term consequences of weight change on depression over certain life periods. Weight change, including weight gain and loss, is also an important factor in physical and mental health.

The Whitehall II Study with participants aged 35–55 years at baseline showed that weight gain was associated with an elevated risk of psychological distress compared with stable weight over the subsequent 10 years (Knüppel et al., 2019). Although some previous studies have reported that weight gain was associated with an increased risk of depression (Ball et al., 2009; Knüppel et al., 2019; Zanardo et al., 2021), the results were not entirely consistent, and some studies have reported no significant associations (Forman-Hoffman et al., 2007; Sahle et al., 2019). In addition, some studies have found that weight loss was also associated with an increased risk of depression (Forman-Hoffman et al., 2007; Knüppel et al., 2019), whereas others have not (Dixon et al., 2003; Sahle et al., 2019). These conflicting results suggested that weight change and depression were closely interrelated and other factors including sex, age, lifestyle, and socioeconomic status could also play significant roles in the association between weight change and depression, which might account for the discrepancies in the results of previous studies.

Excess adiposity tends to accrue during early and middle adulthood for most people. Using data from the National Health and Nutrition Examination Survey (NHANES) 2005–2018, we aimed to examine the relationship between weight changes from young adulthood to midlife and late adulthood with depression.

## 2. Methods

### 2.1. Study design and population

We obtained data from NHANES, a series of ongoing cross-sectional surveys conducted in the United States. Representative samples of the non-institutional US population were selected by a complex stratified, multistage probability sampling design. The survey was approved by the National Center for Health Statistics (NCHS) research ethics review board, and written informed consent from all the participants was provided during the survey.

The NHANES study design has been described in detail previously (Zipf et al., 2013). This study used data across seven continuous cycles of NHANES (2005–2006 through 2017–2018), including adults aged 40–74 at the NHANES survey (Stokes et al., 2018). The study design is shown in Supplementary Figure S1. We excluded participants younger than 40 years or older than 74 years, those without a complete depression screener questionnaire, and those without height or weight information at age 25 years and (or) 10 years before the survey and (or) at the survey.

### 2.2. Assessments of weight change

The survey height and weight of the subjects were measured during physical examination. Participants were asked to recall their weight at age 25 and 10 years before the survey. BMIs at these three-time points were calculated as the corresponding weight (kg) divided by the square of survey height (m^2^). BMI was further categorized into normal weight (<25.0), overweight (25.0–29.9), and obesity (≥30.0). We created weight change patterns for three time intervals: BMI at age 25 to 10 years before the survey, BMI at age 25 to BMI at the survey, and BMI at 10 years before the survey to BMI at the survey. Using BMI at two-time points, we defined five weight change patterns for each interval: stable normal (BMI <25 at both times), maximum overweight (25–29.9 at either time but not ≥30 at the other time), non-obesity to obesity (<30 at younger age and ≥ 30 later), obesity to non-obesity (≥30 at a younger age and < 30 later), and stable obesity (≥30 at both times). To draw a comparison with other studies (Chen et al., 2019; Wang et al., 2021), absolute weight change across the two-time intervals was also classified into five groups: weight loss group (weight loss ≥2.5 kg), stable weight group (weight change within 2.5 kg), small to moderate weight gain (2.5 kg ≤ weight gain <10.0 kg), moderate to large weight gain (10 kg ≤ weight gain <20.0 kg), and extreme weight gain group (weight gain ≥20.0 kg).

### 2.3. Depression

Current depressive symptoms were assessed with the Patient Health Questionnaire-9 (PHQ-9) questionnaire (Patel et al., 2019). The questionnaire was administered by trained interviewers using Computer Assisted Personal Interview technology during Mobile Examination Center visits. The PHQ-9 refers to the previous 2-week interval and consists of nine items on depression symptoms and one follow-up question on functional impairment. Each symptom was scored by a 4-point ascending Likert scale ranging from 0 to 3. If the individual has not suffered the symptom at all over the past 2 weeks, the score is zero, while if the individual suffered the symptom “nearly every day,” the score is 3. Therefore, the summation of total scores on the PHQ-9 may range from 0 to 27. According to previous research, patients with PHQ-9 scores ≥10 were considered at high risk of depression (Jackson et al., 2019). The internal reliability of the PHQ-9 was excellent, with a Cronbach’s α of 0.89 in the PHQ Primary Care Study. Test–retest reliability of the PHQ-9 was also excellent. The correlation between the two measurements of PHQ-9 was 0.84, and the mean scores were nearly identical (5.08 vs. 5.03) (Kroenke et al., 2001).

### 2.4. Covariates

The potential confounding factors, including sex, age at the survey, race/ethnicity, family income-to-poverty ratio (PIR) (U.S. Department of Health & Human Services, 2023), educational level, smoking status, and drinking status were obtained via the demographic questionnaires. Race/ethnicity was grouped into Mexican American, non-Hispanic white, non-Hispanic black, other Hispanic, and others. Educational level was classified as less than high school, high school or equivalent, and college or above. Family PIR was calculated by dividing family income according to the poverty guidelines and further divided into 4 categories (0–1.3, −1.8, −3.0, and > 3.0). Lifestyle factors included alcohol use (Heavy drinker, low to moderate drinker, and non-drinker) and smoking status (never, former, current smoker). A drinker was defined as any participant who had at least 12 drinks of any type of alcoholic beverage in any 1 year.

### 2.5. Statistical analysis

All analyses incorporated sampling weights, strata, and primary sampling units (PSU) to account for the complex sampling design of NHANES and to provide nationally representative estimates. Data for population characteristics are presented as the mean and standard deviation (SD) for continuous variables, while the frequency (*n*) and proportion (%) for categorical variables are presented.

The multivariable logistic regression model was used to explore the independent relationship between weight status or weight change patterns and depression. The stable normal pattern was used as the reference to which all other weight change patterns were compared. The first model (model 1) was unadjusted for potential confounding. We adjusted for survey age and sex in model 2. We further adjusted for race/ethnicity, education level, family income-poverty ratio level, smoking status, and drinking status in model 3. There are 5, 9, 847, and 1,455 participants who had missing information for the covariates smoking status, educational level, drinking status, and PIR, respectively. Dummy variables were used to indicate missing data for the covariates. Predefined subgroup analyses and potential effect modifications were conducted by survey age (< 55 and ≥ 55 years), sex (Male and Female), and smoking status (Ever and Never smoking). No adjustment for multiplicity in the subgroup analysis was done because of the exploratory design of this study part.

We also investigated the associations between absolute weight change groups and depression risk. The absolute weight changes were also treated as continuous variables to examine the robustness of our results. The stable weight group was used as the reference to which all other weight change patterns were compared.

We also assessed the dose–response relationship using a restricted cubic spline with five knots at the 5th, 25th, 50th, 75th, and 95th. The adjusted covariates in the restricted cubic spline were the same as the covariates adjusted in model 2 of the logistic regression.

All statistical analyses were conducted in 2021 using SAS 9.4 (SAS Institute Inc., Cary, NC, United States). The forest plot was made by the “forestplot” package in R 4.0.2 (R Foundation for Statistical Computing, Vienna, Austria). Statistical significance was defined as *p* < 0.05 using two-sided tests.

## 3. Results

### 3.1. Baseline characteristics and weight change pattern

Table 1 reported characteristics of study participants across weight change patterns from early to middle adulthood. The mean age of the sample was 55 years, and 51.4% were female. The study sample was 72.7% non-Hispanic white, 10.4% non-Hispanic black, 6.0% Mexican American, and 4.3% other Hispanic. The mean BMI was 23.7 kg/m^2^ at age 25, 27.8 kg/m^2^ at 10 years before the survey, and 29.6 kg/m^2^ at the survey. On average, participants gained 10.5 kg weight from age 25 years to 10 years before the survey, 5.0 kg in the 10 years period before the survey, and 15.5 kg from age 25 years to the survey (Table 1; Supplementary Tables S1, S2).

**Table 1:** Characteristics of study participants in NHANES 2005–2018 according to weight change patterns from age 25 years to 10 years before survey.^a^

Characteristics	Total	Stable normal	Maximum overweight	Non-obesity to obesity	Obesity to non-obesity	Stable obesity	*p* value
Participants	17,556	5,844 (35.30)	6,347 (35.43)	3,911 (21.32)	178 (0.82)	1,276 (7.12)	
Age (mean ± SD^b^, years)	54.96 ± 0.14	53.49 ± 0.19	55.60 ± 0.19	57.57 ± 0.26	52.20 ± 0.78	51.58 ± 0.33	<0.001
Female	8,849 (51.37)	3,513 (64.55)	2,667 (41.40)	1961 (48.05)	78 (48.81)	630 (45.84)	<0.001
Race/ethnicity							
Mexican American	2,504 (6.01)	610 (4.48)	1,002 (6.63)	666 (6.97)	41 (11.62)	185 (7.04)	<0.001
Other Hispanic	1714 (4.33)	517 (3.97)	720 (5.17)	364 (3.74)	21 (7.47)	92 (3.28)	
Non-Hispanic White	7,424 (72.67)	2,568 (73.60)	2,609 (72.05)	1,679 (73.79)	69 (64.69)	499 (68.65)	
Non-Hispanic Black	4,113 (10.42)	1,213 (9.17)	1,421 (9.88)	1,006 (11.19)	40 (13.41)	433 (16.68)	
Other	1801 (6.57)	936 (8.78)	595 (6.27)	196 (4.32)	7 (2.81)	67 (4.34)	
Education							
Less than high school	4,029 (13.72)	1,221 (12.98)	1,514 (14.18)	939 (13.73)	68 (25.39)	287 (13.71)	<0.001
High school or equivalent	4,101 (23.56)	1,304 (21.57)	1,479 (24.25)	958 (24.87)	33 (20.90)	327 (26.35)	
College or above	9,418 (62.72)	3,314 (65.45)	3,352 (61.57)	2014 (61.41)	77 (53.70)	661 (59.94)	
Family income-poverty ratio level							
0 ~ 1.3	4,336 (15.80)	1,377 (15.13)	1,528 (15.10)	991 (16.39)	78 (34.20)	362 (18.64)	<0.001
~1.85	2032 (8.84)	630 (8.01)	757 (8.82)	469 (9.50)	21 (13.07)	155 (10.61)	
~3	2,811 (16.43)	899 (15.54)	983 (16.41)	694 (17.75)	18 (8.66)	217 (17.87)	
>3	6,922 (58.93)	2,441 (61.31)	2,557 (59.67)	1,435 (56.37)	53 (44.07)	436 (52.89)	
Smoking status							
Never smoker	8,911 (51.27)	2,960 (51.07)	3,233 (51.70)	1985 (50.97)	58 (28.80)	675 (53.51)	<0.001
Former smoker	4,950 (29.20)	1,417 (25.64)	1862 (30.31)	1,286 (33.62)	57 (32.07)	328 (27.74)	
Current smoker	3,690 (19.53)	1,466 (23.29)	1,249 (17.98)	640 (15.41)	63 (39.13)	272 (18.75)	
Drinking status							
Non-drinker	5,123 (23.89)	1,604 (21.94)	1773 (23.00)	1,282 (27.42)	48 (25.98)	416 (27.53)	<0.001
Low to moderate drinker	6,312 (42.27)	2,157 (42.36)	2,316 (42.83)	1,384 (43.40)	49 (29.47)	406 (37.06)	
Heavy drinker	5,274 (33.84)	1853 (35.71)	1961 (34.17)	1,005 (29.19)	68 (44.55)	387 (35.41)	
Body mass index (mean ± SD^b^)							
At age 25 years	23.73 ± 0.06	20.68 ± 0.04	23.59 ± 0.05	24.91 ± 0.08	34.26 ± 0.57	34.70 ± 0.17	<0.001
At 10 years before survey	27.83 ± 0.08	22.29 ± 0.03	27.16 ± 0.03	34.37 ± 0.11	26.65 ± 0.25	39.18 ± 0.27	<0.001
Absolute weight change (mean ± SD^b^, kg)	10.45 ± 0.15	3.82 ± 0.11	9.04 ± 0.15	24.65 ± 0.35	−20.36 ± 1.86	11.30 ± 0.74	<0.001

a All estimates accounted for complex survey designs. Data are expressed as No. (%) unless otherwise indicated.

b SD, Standard deviation.

From age 25 years to 10 years before the survey, 35.3% of the participants were in the stable normal group, 35.4% were in the maximum overweight group, 7.1% were in the stable obesity group, 21.3% of the participants moved from non-obesity to obesity and they gained 24.7 kg on average, whereas only 0.8% of the participants moved from the obesity to non-obesity category and they lost 20.4 kg on average (Table 1). For the 10 years period before the survey, 17.0% of the participants reported gaining weight, and 4.6% reported losing weight from the obesity to non-obesity category (Supplementary Table S1). The corresponding numbers were 33.9% and 1.0%, respectively, from age 25 years to the survey (Supplementary Table S2).

### 3.2. Associations of weight status with depression

Among 17,556 participants, 1,672 had a diagnosis of depression, yielding an overall prevalence rate of 8.0%. When evaluating the weight status at each time point, we found that obesity was significantly associated with depression, with ORs (95% CI) of 1.43 (1.14 to 1.78) at age 25 years, 1.62 (1.35 to 1.93) at 10 years before the survey, and 1.69 (1.39 to 2.07) at the survey (Supplementary Table S3). There were significant linear associations between BMI at the age of 25 years, BMI at 10 years before the survey and depression (*P*_non-linearity_ > 0.05). Whereas the association between BMI at survey and depression changed to a U shape (*P*_non-linearity_ < 0.05) (Supplementary Figure S2).

### 3.3. Associations of weight change patterns with depression

Table 2 showed the association between weight change patterns in the three periods and the risk of depression, using the stable normal group as the reference. As expected, stable obesity participants had increased risks of depression across adulthood, with ORs (95% CI) of 1.61 (1.23 to 2.11) from age 25 years to 10 years before the survey, 2.15 (1.71 to 2.70) in the 10 years period before the survey, and 1.88 (1.44 to 2.44) from age 25 years to the survey. Weight gain from the non-obesity range at age 25 years to the obesity range at 10 years before the survey was associated with a 71% higher risk of depression (OR = 1.71, 95% CI = 1.41 to 2.04), the ORs (95% CI) were 1.73 (1.35 to 2.21) in the 10 years period before the survey and 1.83 (1.49 to 2.24) for the period from age 25 years to survey. The maximum overweight group also showed significant associations with depression risk, with ORs (95% CI) of 1.27 (1.08 to 1.49), 1.39 (1.11 to 1.74), and 1.27 (1.01 to 1.59). Individuals who reported losing weight from the obesity category to the non-obesity group had null associations in all three-time intervals, with ORs (95% CI) of 1.74 (0.93 to 3.23), 1.40 (1.00 to 1.95), and 1.58 (0.92 to 2.73).

**Table 2:** Odd ratios (OR) and 95% confidence intervals (CIs) of depression with weight change patterns across adulthood in the NHANES 2005–2018.^a^

Weight change patterns^b^	No. of depression/No. of participants	Model 1^c^		Model 2^d^		Model 3^e^	
		OR (95% CI)	*p*	OR (95% CI)	*p*	OR (95% CI)	*p*
From age 25 years to 10 years before survey							
Stable normal (ref)	482/5844	1.00		1.00		1.00	
Maximum overweight	549/6347	1.04 (0.90, 1.21)	0.582	1.23 (1.05, 1.43)	0.009	1.27 (1.08, 1.49)	0.004
Non-obesity to obesity	450/3911	1.44 (1.20, 1.72)	<0.001	1.65 (1.38, 1.98)	<0.001	1.71 (1.42, 2.04)	<0.001
Obesity to non-obesity	27/178	2.40 (1.40, 4.13)	0.002	2.62 (1.51, 4.54)	0.001	1.74 (0.93, 3.23)	0.080
Stable obesity	164/1276	1.49 (1.14, 1.94)	0.003	1.64 (1.26, 2.13)	<0.001	1.61 (1.23, 2.11)	0.001
From 10 years before survey to survey							
Stable normal (ref)	243/3389	1.00		1.00		1.00	
Maximum overweight	447/5852	1.19 (0.95, 1.49)	0.120	1.36 (1.09, 1.70)	0.008	1.39 (1.11, 1.74)	0.004
Non-obesity to obesity	368/3128	1.68 (1.33, 2.13)	<0.001	1.76 (1.39, 2.23)	<0.001	1.73 (1.35, 2.21)	<0.001
Obesity to non-obesity	101/928	1.44 (1.04, 2.00)	0.030	1.69 (1.21, 2.36)	0.002	1.40 (1.00, 1.95)	0.051
Stable obesity	513/4259	1.81 (1.43, 2.28)	<0.001	2.02 (1.60, 2.55)	<0.001	2.15 (1.71, 2.70)	<0.001
From age 25 years to survey							
Stable normal (ref)	284/3822	1.00		1.00		1.00	
Maximum overweight	475/6108	1.09 (0.87, 1.36)	0.443	1.22 (0.98, 1.53)	0.076	1.27 (1.01, 1.59)	0.038
Non-obesity to obesity	722/6172	1.62 (1.32, 1.98)	<0.001	1.73 (1.41, 2.13)	<0.001	1.83 (1.49, 2.24)	<0.001
Obesity to non-obesity	32/239	2.00 (1.25, 3.23)	0.005	2.32 (1.42, 3.78)	0.001	1.58 (0.92, 2.73)	0.098
Stable obesity	159/1215	1.75 (1.34, 2.29)	<0.001	1.86 (1.43, 2.42)	<0.001	1.88 (1.44, 2.44)	<0.001

a All estimates accounted for complex survey designs.

b Stable normal pattern (<25.0 at both times), maximum overweight pattern (25.0–29.9 at either time but not ≥30.0 at the other time), non-obesity to obesity pattern (<30.0 at younger age and ≥ 30.0 later), obesity to non-obesity pattern (≥30.0 at younger age and < 30.0 later), and stable obesity (≥30.0 at both times).

c Model 1 was unadjusted.

d Model 2 was adjusted for age and sex.

e Model 3 was additionally adjusted for race/ethnicity, education level, family income-poverty ratio level, smoking status, and drinking status.

**Figure 1:**
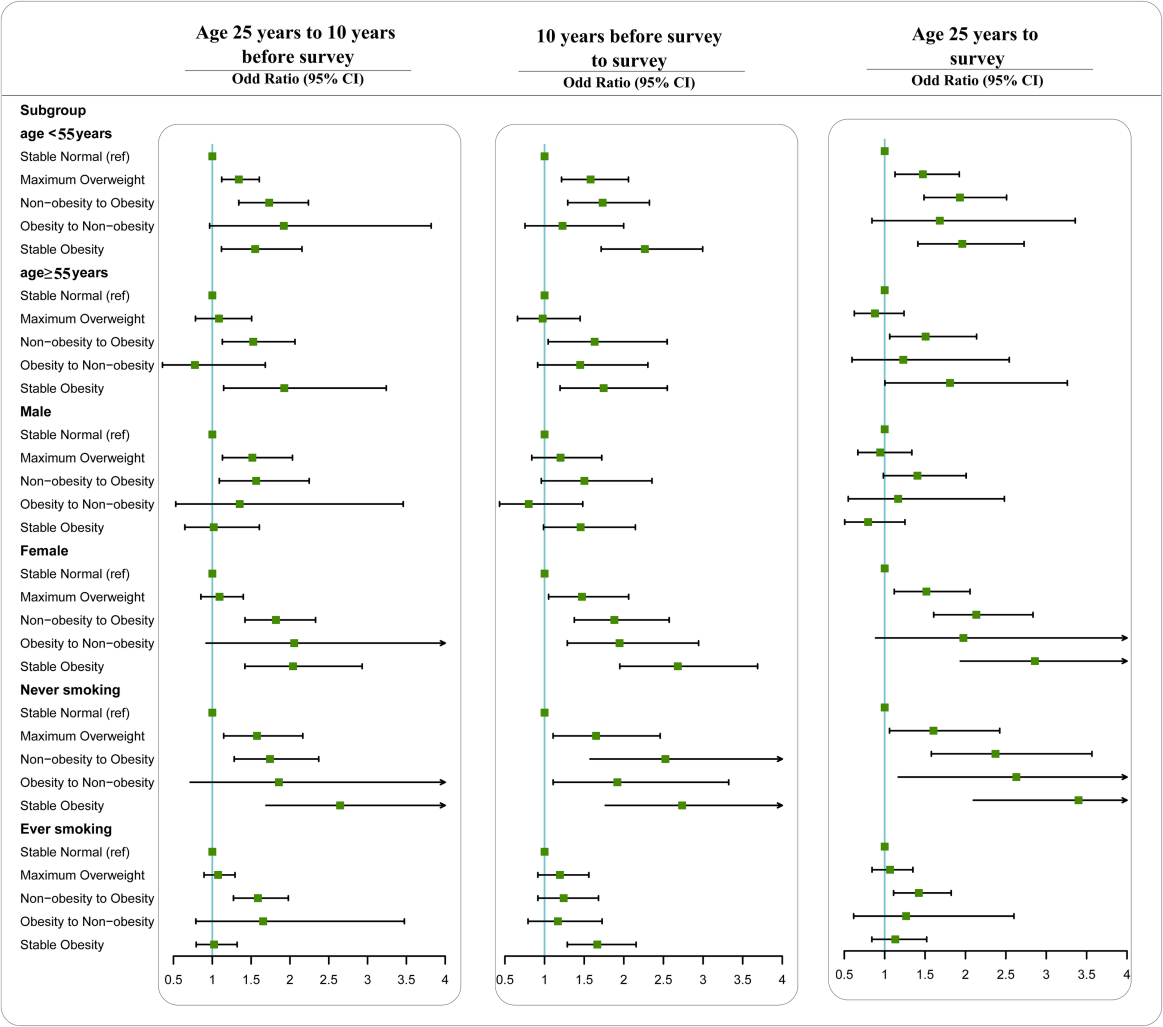
Associations between weight change patterns across adulthood and risk of depression stratified by age, sex, and smoking status in NHANES 2005–2018. All estimates accounted for complex survey design of NHANES. Risk estimates were adjusted for age (not adjusted in subgroup analysis by age), sex (not adjusted in subgroup analysis by sex), race/ethnicity, education level, family income-poverty ratio level, smoking status (not adjusted in subgroup analysis by smoking status), and drinking status.

In the stratified analyses, we found significant interactions with sex and smoking status but not with age (Figure 1). In the analysis of weight change from age 25 years to 10 years before the survey, the effects of stable obesity on depression were more pronounced in females (OR = 2.04, 95% CI = 1.42 to 2.93) than in males (OR = 1.02, 95% CI = 0.65 to 1.611), *P* for interaction was 0.024. The associations between stable obesity and depression were stronger among never-smokers (OR = 1.75, 95% CI = 1.43 to 2.15) compared with ever-smokers (OR = 1.11, 95% CI = 0.57 to 2.18), *P* for interaction was <0.001. We found similar results according to the weight change pattern in the other two time periods.

**Figure 2:**
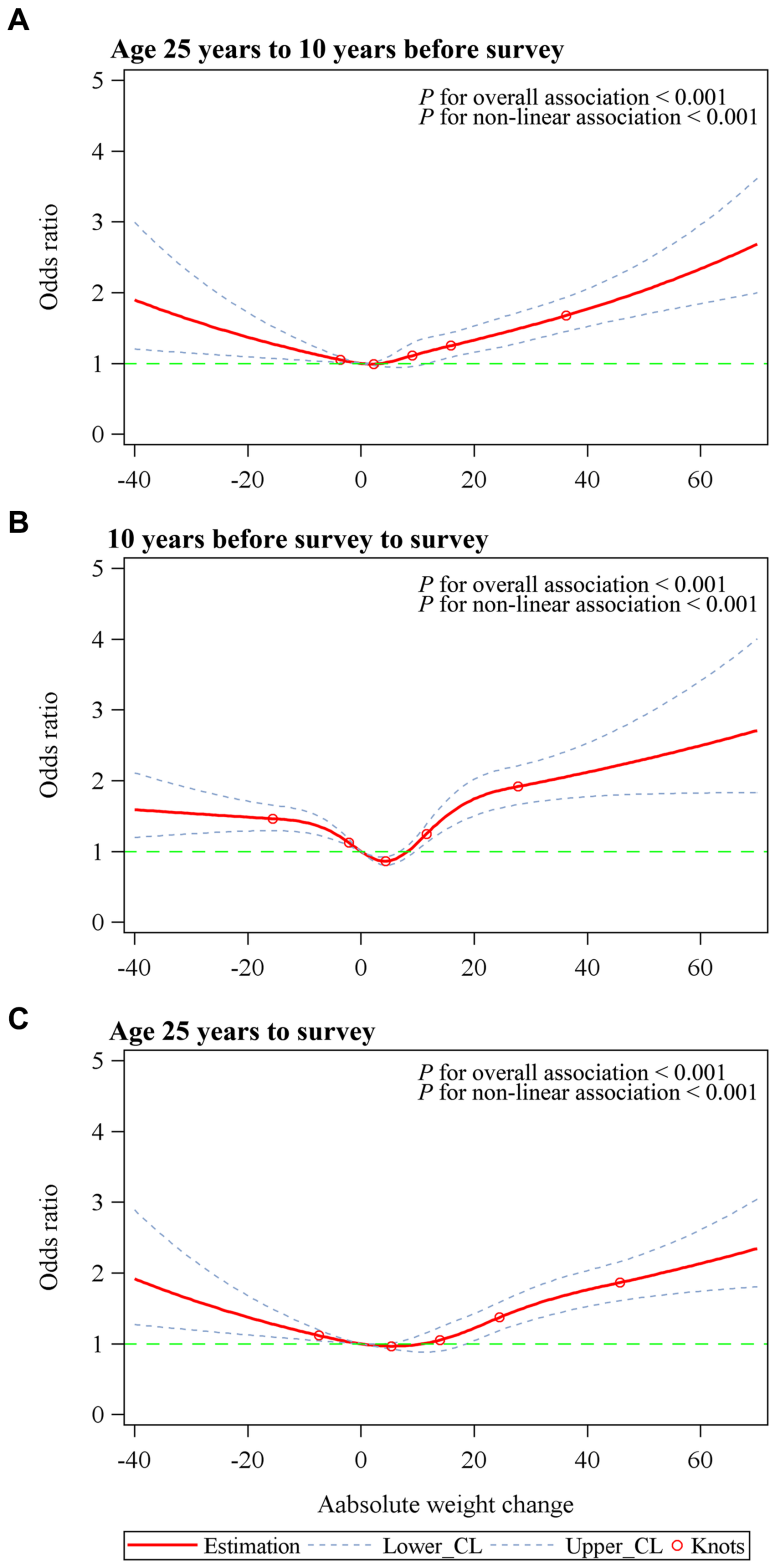
Dose–response association between absolute weight change across adulthood and risk of depression. **(A)** Age 25 years to 10 years before survey. **(B)** 10 years before survey to survey. **(C)** Age 25 years to survey. Associations were examined by multivariable logistic regression models based on restricted cubic splines. Red solid line represents estimates of odds ratios (reference value for absolute weight change: 0 kg). Blue dashed line represents 95% confidence intervals. Red circle represents knots located at the 5th, 25th, 50th, 75th and 95th percentiles of the distribution of absolute weight change. Risk estimates were adjusted for age, sex, race/ethnicity, education level, family income-poverty ratio level, smoking status, and drinking status. For weight change from age 25 years to 10 years before survey or to survey, BMI at age 25 was also adjusted for. For weight change from 10 years before survey to survey, BMI at 10 years previously was also adjusted for. *p* values for overall association and *p* values for non-linear association were all <0.001 in three periods.

When evaluating the absolute weight changes, we identified a J-shaped or U-shaped association for depression with weight change across the three time intervals (Figure 2). When classified into categories, the ORs (95% CI) for depression in the extreme weight gain group (weight gain ≥20 kg) were 1.60 (1.28 to 2.00) from age 25 years to 10 years before the survey, 2.01 (1.53 to 2.63) in the 10 years period before the survey, and 1.86 (1.32 to 2.63) from age 25 years to survey, compared with the stable weight group (weight change within 2.5 kg) (Table 3). Moderate to large weight gain (weight gain ≥10 kg and < 20 kg) also showed significant associations with depression risk. Small to moderate weight gain (weight gain ≥2.5 kg and < 10 kg) was not significantly associated with depression in any of the three-time intervals. Participants who lost more than 2.5 kg in the 10 years period before the survey had an OR (95% CI) of 1.54 (1.17 to 2.02) for depression, with an OR (95% CI) of 1.50 (1.02 to 2.21) from age 25 years to the survey, whereas the association was not significant from age 25 years to 10 years before the survey (OR = 0.99, 95% CI = 0.72 to 1.35). When we restricted the analysis to participants who had a BMI of <25 at 25 years of age or at 10 years before survey, results remained similar, suggesting that our estimates were not driven by participants with high BMIs who gained additional weight (Supplementary Figure S3). When stratified by age, sex, and smoking status, the associations were similar to our main results (Supplementary Figure S3).

**Table 3:** Odd ratios (OR) and 95% confidence intervals (CIs) of depression with absolute weight change groups across adulthood in the NHANES 2005–2018.^a^

Weight change patterns ^b^	No. of depression/No. of participants	Model 1^c^		Model 2^d^		Model 3^e^	
		OR (95% CI)	*p*	OR (95% CI)	*p*	OR (95% CI)	*p*
From age 25 years to 10 years before survey							
Weight loss ≥2.5 kg	120/964	1.29 (0.97, 1.71)	0.081	1.31 (0.98, 1.75)	0.071	0.99 (0.72, 1.35)	0.938
Weight change within 2.5 kg (ref)	342/3898			1.00		1.00	
Weight gain ≥2.5 kg and < 10 kg	435/5398	0.77 (0.63, 0.95)	0.015	0.79 (0.64, 0.98)	0.032	0.93 (0.75, 1.15)	0.492
Weight gain ≥10 kg and < 20 kg	380/4030	1.09 (0.87, 1.36)	0.449	1.14 (0.90, 1.45)	0.272	1.30 (1.02, 1.66)	0.036
Weight gain ≥20 kg	395/3266	1.45 (1.19, 1.76)	<0.001	1.52 (1.22, 1.88)	<0.001	1.60 (1.28, 2.00)	<0.001
From 10 years before survey to survey							
Weight loss ≥2.5 kg	507/4209	2.27 (1.78, 2.90)	<0.001	2.31 (1.80, 2.96)	<0.001	1.54 (1.17, 2.02)	0.003
Weight change within 2.5 kg (ref)	192/3224			1.00		1.00	
Weight gain ≥2.5 kg and < 10 kg	339/4982	1.17 (0.93, 1.47)	0.181	1.12 (0.89, 1.41)	0.317	1.10 (0.87, 1.39)	0.409
Weight gain ≥10 kg and < 20 kg	349/3246	1.97 (1.52, 2.55)	<0.001	1.82 (1.41, 2.35)	<0.001	1.60 (1.22, 2.09)	0.001
Weight gain ≥20 kg	285/1895	2.88 (2.23, 3.71)	<0.001	2.56 (2.00, 3.29)	<0.001	2.01 (1.53, 2.63)	<0.001
From age 25 years to survey							
Weight loss ≥2.5 kg	216/1697	2.14 (1.49, 3.09)	<0.001	2.22 (1.54, 3.20)	<0.001	1.50 (1.02, 2.21)	0.039
Weight change within 2.5 kg (ref)	101/1442			1.00		1.00	
Weight gain ≥2.5 kg and < 10 kg	246/3601	0.91 (0.66, 1.26)	0.562	0.89 (0.65, 1.23)	0.481	0.95 (0.68, 1.33)	0.759
Weight gain ≥10 kg and < 20 kg	390/4822	1.18 (0.87, 1.62)	0.285	1.15 (0.84, 1.57)	0.368	1.19 (0.86, 1.65)	0.281
Weight gain ≥20 kg	719/5994	1.96 (1.40, 2.73)	<0.001	1.85 (1.32, 2.57)	<0.001	1.86 (1.32, 2.63)	0.001

a All estimates accounted for complex survey designs.

b Absolute weight change: weight loss of at least 2.5 kg, weight change within 2.5 kg, weight gain of at least 2.5 kg but less than 10.0 kg, weight gain of at least 10 kg but less than 20.0 kg, and weight gain of at least 20.0 kg.

c Model 1 was unadjusted.

d Model 2 was adjusted for age and sex.

e Model 3 was additionally adjusted for race/ethnicity, education level, family income-poverty ratio level, smoking status, and drinking status.

## 4. Discussion

In this large, nationally representative survey of US adults, we found that the lowest risk of depression was in the stable normal participants, whereas both stable obesity and weight gain across adulthood were associated with increased risks of depression. The effects of weight change patterns on the risks of depression were more pronounced in females than in males. Our findings underscore that it was important to maintain a normal weight throughout adulthood, especially the prevention of stable obesity and weight gain in late adulthood, for reducing the risk of depression.

The relationship between BMI and depression has been extensively investigated in many studies. Consistent with previous studies, our study suggested that obesity was significantly associated with an increased risk of depression using BMI at 25 years, at 10 years before the survey and the survey. In addition, we found that obesity increased the risk for depression in a dose–response manner, and this finding was consistent with a previous meta-analysis (Jung et al., 2017). The results of our study showed a U-shaped association between BMI at the survey and risk of depression. Several studies (Noh et al., 2015; Lee et al., 2017) have also indicated a U-shaped association between BMI and levels of depressive symptoms among adults. The risk of depression was higher both in the obese and underweight participants. Future studies are warranted to explore the underlying mechanism between BMI and depression.

Weight changes are common across adulthood, thus recently more studies focused on the relationship between weight changes and health outcomes (Lee et al., 2020; Bae et al., 2021). A cohort study of 17,522 Britons with repeated measures of BMI reported that weight change was associated with an increased risk of depression in mid-aged and elderly people. Weight gain was associated with 20% increase in the risk of depression (Knüppel et al., 2019). In a study conducted in Australia (Williams et al., 2006), small weight gain (1.5%–2.5%) increased the risk of depression by 14% and 18%, respectively. High weight gain (>2.5%) in the first 3 years of baseline was associated with a 30% increased risk of depression. Data from the Korean National Health and Nutrition Examination Survey (2014, 2016, 2018) suggested that in relatively healthy middle-aged Korean women (Jung et al., 2021), weight change was a significant factor associated with mental health. Thus, our results are consistent with these studies, suggesting that avoiding obesity in young adulthood and preventing weight gain from young to late adulthood could be a crucial approach to reduce the risk of future depression.

There are many plausible mechanisms for the association between obesity and the risk of depression. Firstly, the hypothalamic–pituitary–adrenal (HPA) axis hyperactivation represents a potentially relevant mechanism between obesity and depression (Milaneschi et al., 2019). HPA axis hyperactivation can be found in nearly half of adult obese persons (Wester et al., 2014), leading to a nonadaptive unabated release of cortisol. Long-term exposure to cortisol can result in neuronal damage and loss in limbic regions vulnerable to stress and associated with depression. Second, chronic immuno-inflammatory activation is another possible underlying mechanism between obesity and depression (Milaneschi et al., 2019). Inflammatory markers such as interleukin-6 (IL-6) and C-reactive protein (CRP), synthesized by adipocytes, are related to depression-related symptoms (Tilg and Moschen, 2006). Other biological mechanisms, such as neuroendocrine changes in the leptin-melanocortin pathway and overlapping genetic variation (e.g., OLFM4 and NEGR1), could also explain the correlation between obesity and depression. Further studies are needed to elucidate the potential mechanisms.

The association between weight change and the risk of depression showed sex disparities. Subgroup analyses by sex indicated that the significant associations between weight change and depression were only found in females. Previous studies that analyzed the relationship between obesity and depression also revealed that it differed by sex (Ul-Haq et al., 2014; Mutz and Lewis, 2021). It seemed that the current ideal of thinness affected females more than their male counterparts and caused more psychological distress in females, which could, in turn, lead to depression. A sexual dimorphism existed with body composition, in those females carried more fat subcutaneously and males carried more fat viscerally (Larsson et al., 2015). This led to distinct differences in the inflammatory pattern exhibited. For example, leptin was secreted more highly from subcutaneous adipose tissue and was therefore higher in females than males (Wang et al., 2021).

Among the obese participants, our study found that weight loss was not significantly associated with the risk of depression compared with those who were stable obese. However, a previous meta-analysis (Cameron et al., 2019) indicated that obese individuals in weight loss trials experienced reductions in symptoms of depression. In our study, we could not differentiate intentional from unintentional weight change among the participants. In the future studies, more information about the reasons of weight change may enable better interpretation of the association between weight change and the risk of depression. On the other hand, the sample size (only 0.82% of the participants switched from obesity to non-obesity from young to late adulthood) was relatively small, and this might be the reason that we detected a marginally significant association between obesity to non-obesity participants (Xie et al., 2016).

The current study had several strengths, especially the nationally representative survey, large sample size and detailed analysis of weight change from young adulthood through midlife to late adulthood. The participants of NHANES were selected from the general, non-institutionalized population, thus our results could be largely generalizable. In addition, the detailed data collection in the NHANES allowed us to adjust for several important confounders that might influence the risk of depression.

Several limitations should be noted in the current study. Firstly, we used recalled and self-reported weight data at the age of 25 and 10 years before the NHANES survey, therefore misclassification bias might have been brought in. Nonetheless, through some validation studies and a recent meta-analysis (De Rubeis et al., 2019), we can still hold that recalled weight in early life might be an effective method for epidemiological analysis in the life course. Secondly, the depressive symptoms were evaluated by the PHQ-9 but not the depression of clinical diagnosis. This tool could not give information about depression period and if some change had been occurred related to life events. However, PHQ-9 showed good reliability and validity, and high adaptability for patients with depression (Kroenke et al., 2001). Finally, due to the lack of data at different time points, we did not assess the relationship between other obesity-related body markers, such as changes in waist circumference and fat mass, and depression. Further studies with repeated data for these body markers may provide a more thorough outlook of changes in weight status and depression risk.

## 5. Conclusion

Our study, using a representative sample of the US population, suggested that weight changes throughout adulthood were associated with an increased risk of depression. These results needed to be confirmed and further explored in prospective cohort studies. Besides, future studies are needed to reveal the mechanisms underlying the association between weight changes and the risk of depression throughout adulthood. Taken together, our findings suggested that maintaining normal weight throughout adulthood, especially prevention of stable obesity and weight gain across adulthood, should be encouraged to reduce the risk of depression. Therefore, monitoring weight change since young adulthood could provide a sensitive and useful clinical measure for early detection of adverse trends in depression risk.

## Data availability statement

Publicly available datasets were analyzed in this study. This data can be found at: https://wwwn.cdc.gov/nchs/nhanes/Default.aspx.

## Ethics statement

Ethical review and approval was not required for the study on human participants in accordance with the local legislation and institutional requirements. Written informed consent to participate in this study was provided by the participants’ legal guardian/next of kin.

## Author contributions

TW and YZ contributed to conception and design of the study. TW, HL, and KF performed the data collection, management and analyses. TW wrote the first draft of the manuscript. BD and HS wrote sections of the manuscript. BD, DZ, and YZ critically edited the manuscript. All authors contributed to the article and approved the submitted version.

## Funding

This work was supported by the Shandong Provincial Natural Science Foundation (ZR2020QG059); Postdoctoral Science Foundation of China (2019M660161); and Qingdao Social Science Planning Research Project (QDSKL2201075).

## Conflict of interest

The authors declare that the research was conducted in the absence of any commercial or financial relationships that could be construed as a potential conflict of interest.

## Publisher’s note

All claims expressed in this article are solely those of the authors and do not necessarily represent those of their affiliated organizations, or those of the publisher, the editors and the reviewers. Any product that may be evaluated in this article, or claim that may be made by its manufacturer, is not guaranteed or endorsed by the publisher.
