# Supplementary material for: Weight change across adulthood in relation to the risk of depression
Source: Front Psychol. 2023 Aug 9;14:1108093. doi: 10.3389/fpsyg.2023.1108093 (PMC10446764; doi:10.3389/fpsyg.2023.1108093)
Supplement: Supplementary file 1 [file Data_Sheet_1.docx]

Supplementary Material

Weight change across adulthood in relation to the risk of depression

Tao Wang^1,2†^, Bingqin Dai^3†^, Huanchen Shi^4†^, Huawei Li^5^, Kexin Fan^5^, Dongfeng Zhang^6^, Yunping Zhou^5*^

*** Correspondence:** Yunping Zhou: lwzhouyunping@163.com

**Figure S1.** **A flow chart of inclusion and exclusion of study participants.**

**
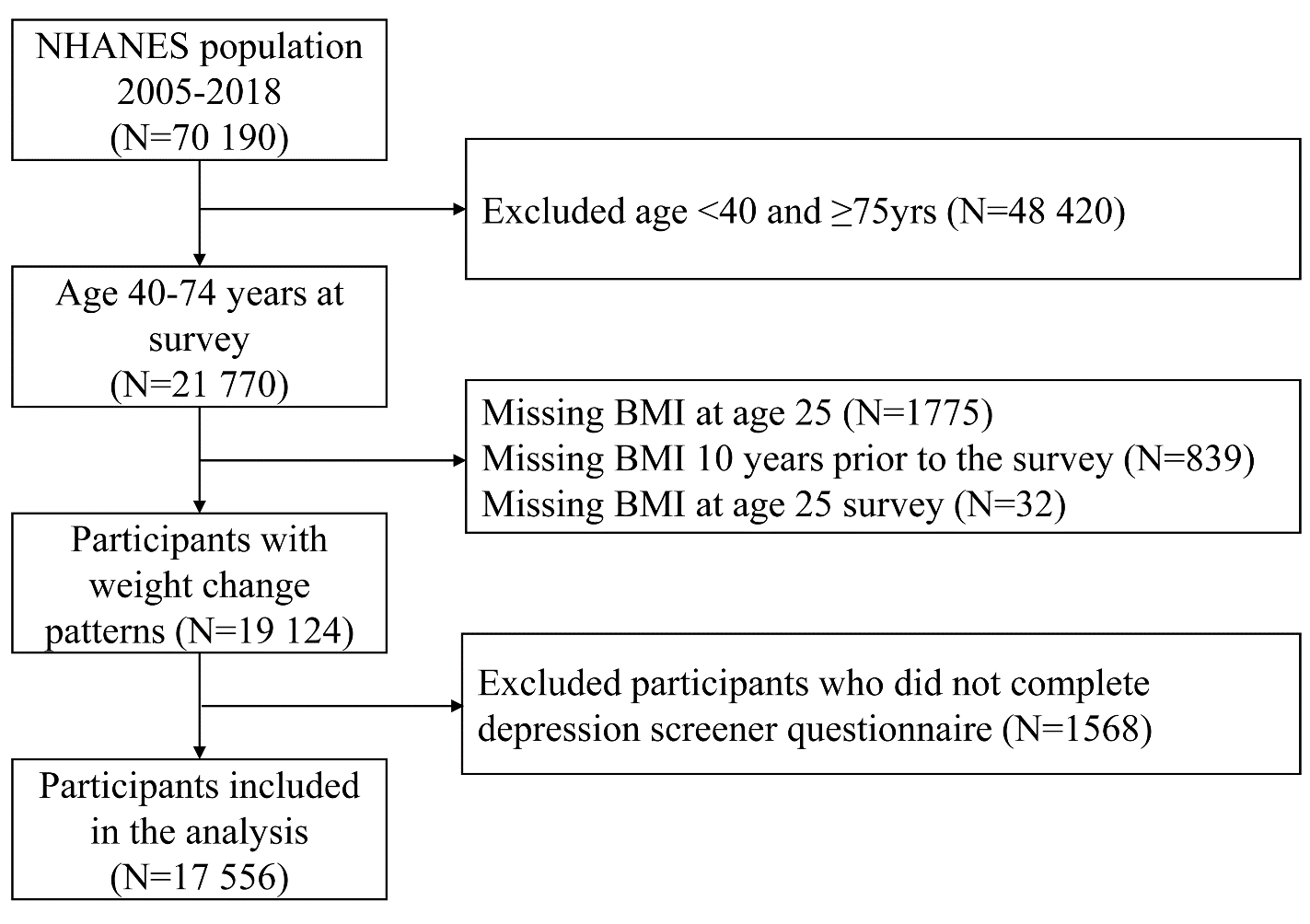
**

**Figure S2. Dose-response association between BMI and risk of depression.** Associations were examined by multivariable logistic regression models based on restricted cubic splines. Red solid line represents estimates of odds ratios (reference value for BMI: 25 kg/m^2^). Blue dashed line represents 95% confidence intervals. Red circle represents knots located at the 5th, 25th, 50th, 75th and 95th percentiles of the distribution of BMI. Risk estimates were adjusted for age, sex, race/ethnicity, education level, family income-poverty ratio level, smoking status, and drinking status.


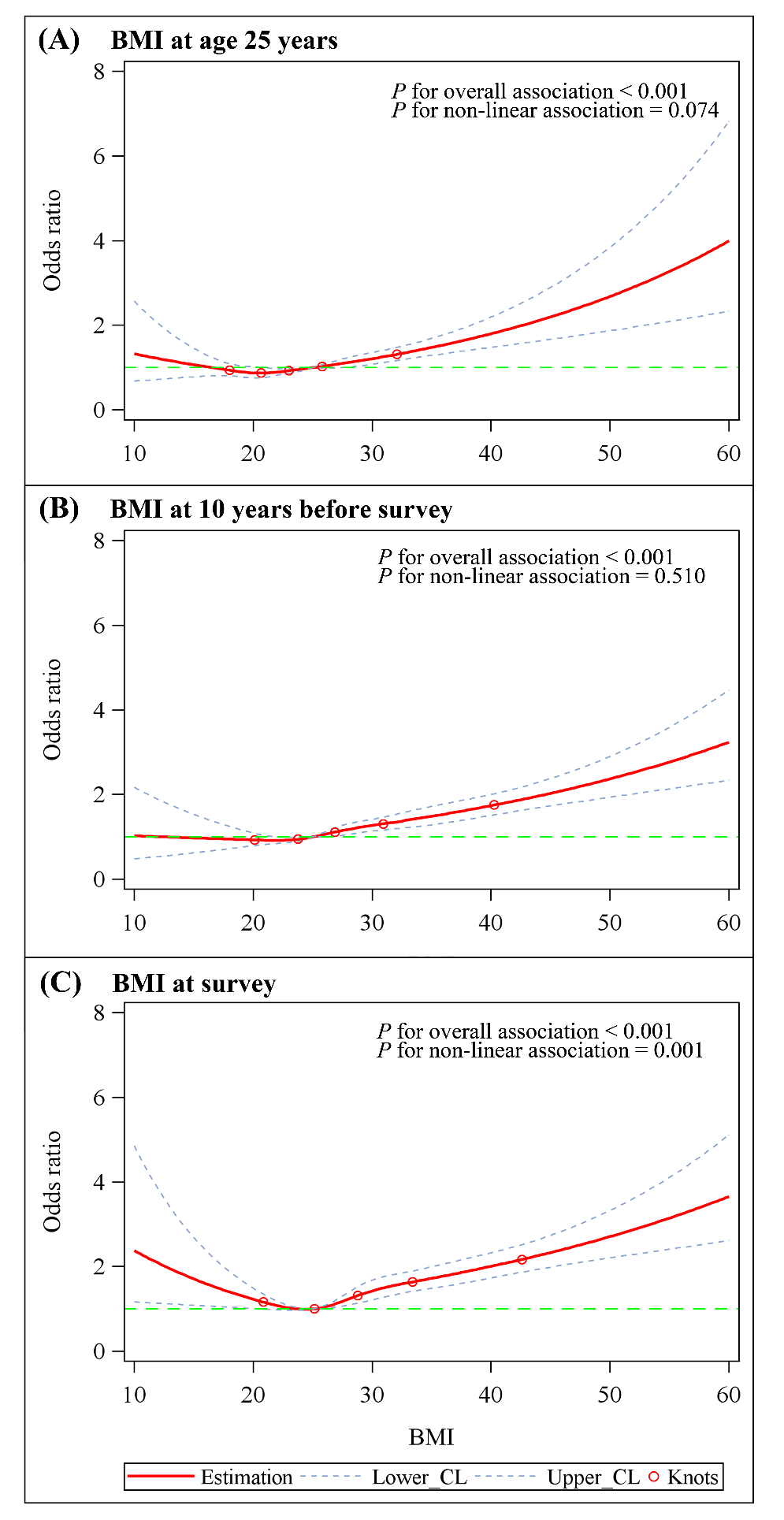


**Figure S3. Associations between absolute weight change groups across adulthood and risk of depression stratified by age, sex, smoking status and weight status in NHANES 2005-2018.** All estimates accounted for complex survey design of NHANES. Risk estimates were adjusted for age (not adjusted in subgroup analysis by age), sex (not adjusted in subgroup analysis by sex), race/ethnicity, education level, family income-poverty ratio level, smoking status (not adjusted in subgroup analysis by smoking status), drinking status, and BMI at 25 years or at 10 years before survey (not adjusted in subgroup analysis by weight status).


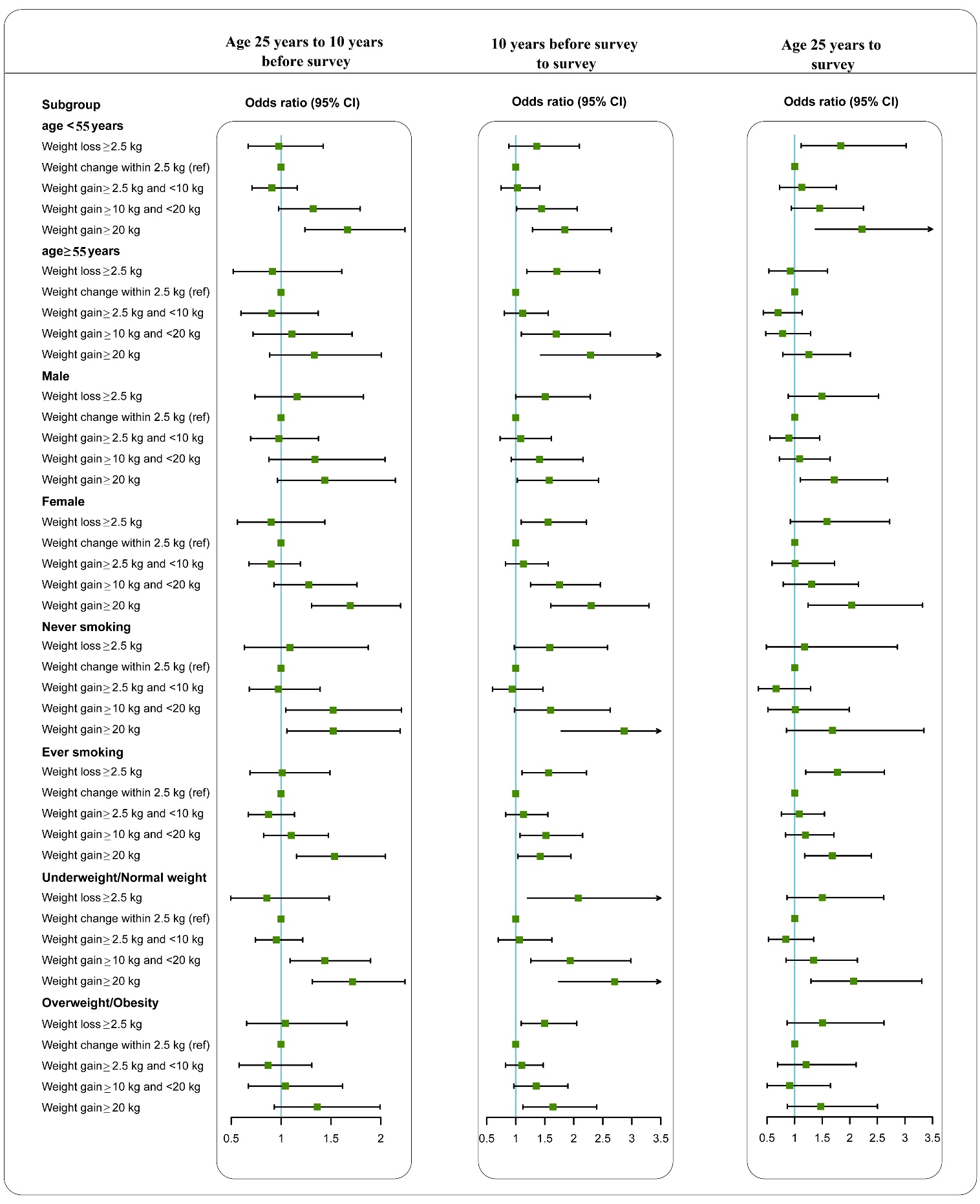


**Table S1. Characteristics of study participants according to weight change patterns from 10 years before survey to survey. ^a^**

| **Characteristics** | **Total** | **Stable Normal** | **Maximum Overweight** | **Non-obesity to Obesity** | **Obesity to Non-obesity** | **Stable Obesity** | ***P* value** |
| --- | --- | --- | --- | --- | --- | --- | --- |
| **Participants** | 17556 | 3389 (20.85) | 5852 (33.72) | 3128 (16.99) | 928 (4.58) | 4259 (23.87) |  |
| **Age (mean ± SD ^b^, years)** | 54.96 ± 0.14 | 54.06 ± 0.23 | 55.07 ± 0.19 | 54.00 ± 0.24 | 57.24 ± 0.53 | 55.85 ± 0.20 | <0.001 |
| **Female** | 8849 (51.37) | 1951 (64.15) | 2463 (43.74) | 1844 (57.33) | 387 (41.95) | 2204 (48.56) | <0.001 |
| **Race/ethnicity** |  |  |  |  |  |  |  |
| Mexican American | 2504 (6.01) | 249 (2.99) | 900 (6.31) | 504 (7.50) | 184 (8.40) | 667 (6.71) | <0.001 |
| Other Hispanic | 1714 (4.33) | 253 (3.29) | 634 (4.91) | 371 (5.61) | 105 (4.96) | 351 (3.37) |  |
| Non-Hispanic White | 7424 (72.67) | 1541 (75.26) | 2489 (73.23) | 1216 (68.64) | 363 (71.04) | 1815 (72.79) |  |
| Non-Hispanic Black | 4113 (10.42) | 642 (7.60) | 1171 (8.77) | 861 (13.58) | 213 (10.99) | 1226 (12.87) |  |
| Other | 1801 (6.57) | 704 (10.86) | 658 (6.78) | 176 (4.67) | 63 (4.61) | 200 (4.27) |  |
| **Education** |  |  |  |  |  |  |  |
| Less than high school | 4029 (13.72) | 659 (11.67) | 1380 (14.33) | 764 (15.02) | 283 (17.78) | 943 (12.95) | <0.001 |
| High school or equivalent | 4101 (23.56) | 745 (20.39) | 1318 (22.90) | 753 (25.94) | 230 (24.81) | 1055 (25.32) |  |
| College or above | 9418 (62.72) | 1982 (67.94) | 3152 (62.77) | 1609 (59.04) | 415 (57.41) | 2260 (61.73) |  |
| **Family income-poverty ratio level** |  |  |  |  |  |  |  |
| 0~1.3 | 4336 (15.80) | 761 (13.69) | 1361 (14.54) | 861 (18.92) | 309 (23.30) | 1044 (15.74) | <0.001 |
| ~1.85 | 2032 (8.84) | 346 (7.49) | 672 (8.38) | 390 (9.87) | 113 (9.86) | 511 (9.75) |  |
| ~3 | 2811 (16.43) | 494 (14.44) | 942 (16.40) | 464 (16.66) | 145 (15.99) | 766 (18.12) |  |
| >3 | 6922 (58.93) | 1502 (64.39) | 2393 (60.68) | 1156 (54.56) | 263 (50.85) | 1608 (56.39) |  |
| **Smoking status** |  |  |  |  |  |  |  |
| Never smoker | 8911 (51.27) | 1711 (51.75) | 2919 (50.78) | 1621 (51.06) | 403 (46.13) | 2257 (52.66) | <0.001 |
| Former smoker | 4950 (29.20) | 731 (22.64) | 1683 (29.60) | 922 (31.52) | 272 (28.88) | 1342 (32.77) |  |
| Current smoker | 3690 (19.53) | 946 (25.61) | 1249 (19.62) | 583 (17.42) | 252 (24.99) | 660 (14.57) |  |
| **Drinking status** |  |  |  |  |  |  |  |
| Non-drinker | 5123 (23.89) | 878 (20.21) | 1570 (22.03) | 977 (26.25) | 309 (27.66) | 1389 (27.40) | <0.001 |
| Low to moderate drinker | 6312 (42.27) | 1339 (44.64) | 2194 (43.57) | 989 (37.56) | 288 (40.19) | 1502 (42.11) |  |
| Heavy drinker | 5274 (33.84) | 1037 (35.15) | 1812 (34.40) | 1033 (36.19) | 276 (32.15) | 1116 (30.48) |  |
| **Body mass index (mean ± SD ^b^)** |  |  |  |  |  |  |  |
| At 10 years before survey | 27.83 ± 0.08 | 21.67 ± 0.04 | 25.54 ± 0.04 | 26.97 ± 0.06 | 33.20 ± 0.18 | 36.03 ± 0.14 | <0.001 |
| At survey | 29.64 ± 0.09 | 22.19 ± 0.04 | 26.95 ± 0.04 | 33.72 ± 0.09 | 27.52 ± 0.10 | 37.46 ± 0.12 | <0.001 |
| **Absolute weight change (mean ± SD ^b^, kg)** | 5.04 ± 0.14 | 1.41 ± 0.12 | 3.95 ± 0.13 | 18.76 ± 0.30 | -16.25 ± 0.60 | 4.08 ± 0.38 | <0.001 |

^a^ All estimates accounted for complex survey designs. Data are expressed as No. (%) unless otherwise indicated.

^b^ SD, Standard Deviation.

**Table S2. Characteristics of study participants according to weight change patterns from age 25 years to survey. ^a^**

| **Characteristics** | **Total** | **Stable Normal** | **Maximum Overweight** | **Non-obesity to Obesity** | **Obesity to Non-obesity** | **Stable Obesity** | ***P* value** |
| --- | --- | --- | --- | --- | --- | --- | --- |
| **Participants** | 17556 | 3822 (23.18) | 6108 (34.93) | 6172 (33.94) | 239 (1.03) | 1215 (6.91) |  |
| **Age (mean ± SD ^b^, years)** | 54.96 ± 0.14 | 54.51 ± 0.24 | 55.18 ± 0.20 | 55.83 ± 0.20 | 53.40 ± 0.69 | 51.38 ± 0.35 | <0.001 |
| **Female** | 8849 (51.37) | 2144 (62.52) | 2579 (43.42) | 3418 (53.15) | 78 (36.80) | 630 (47.53) | <0.001 |
| **Race/ethnicity** |  |  |  |  |  |  |  |
| Mexican American | 2504 (6.01) | 298 (3.12) | 984 (6.60) | 996 (7.02) | 51 (10.14) | 175 (7.12) | <0.001 |
| Other Hispanic | 1714 (4.33) | 310 (3.47) | 651 (4.83) | 640 (4.53) | 31 (7.43) | 82 (3.16) |  |
| Non-Hispanic White | 7424 (72.67) | 1739 (75.24) | 2572 (73.14) | 2545 (71.46) | 82 (62.33) | 486 (69.13) |  |
| Non-Hispanic Black | 4113 (10.42) | 705 (7.47) | 1261 (9.05) | 1674 (12.46) | 60 (14.65) | 413 (16.60) |  |
| Other | 1801 (6.57) | 770 (10.69) | 640 (6.37) | 317 (4.53) | 15 (5.45) | 59 (4.00) |  |
| **Education** |  |  |  |  |  |  |  |
| Less than high school | 4029 (13.72) | 770 (12.07) | 1454 (14.28) | 1450 (13.98) | 98 (28.14) | 257 (12.95) | <0.001 |
| High school or equivalent | 4101 (23.56) | 820 (19.97) | 1431 (23.74) | 1490 (25.30) | 42 (18.00) | 318 (26.95) |  |
| College or above | 9418 (62.72) | 2228 (67.96) | 3222 (61.98) | 3230 (60.72) | 99 (53.86) | 639 (60.10) |  |
| **Family income-poverty ratio level** |  |  |  |  |  |  |  |
| 0~1.3 | 4336 (15.80) | 870 (13.94) | 1461 (15.05) | 1565 (16.76) | 100 (31.75) | 340 (18.57) | <0.001 |
| ~1.85 | 2032 (8.84) | 415 (7.92) | 683 (8.20) | 758 (9.65) | 33 (12.99) | 143 (10.55) |  |
| ~3 | 2811 (16.43) | 576 (15.17) | 975 (16.13) | 1025 (17.49) | 30 (12.05) | 205 (17.61) |  |
| >3 | 6922 (58.93) | 1645 (62.97) | 2452 (60.61) | 2336 (56.10) | 61 (43.21) | 428 (53.27) |  |
| **Smoking status** |  |  |  |  |  |  |  |
| Never smoker | 8911 (51.27) | 1898 (50.86) | 3058 (51.15) | 3222 (51.73) | 77 (35.28) | 656 (53.30) | <0.001 |
| Former smoker | 4950 (29.20) | 849 (22.96) | 1771 (29.76) | 1945 (33.12) | 66 (29.70) | 319 (27.96) |  |
| Current smoker | 3690 (19.53) | 1074 (26.18) | 1278 (19.09) | 1003 (15.14) | 95 (35.01) | 240 (18.74) |  |
| **Drinking status** |  |  |  |  |  |  |  |
| Non-drinker | 5123 (23.89) | 1011 (20.69) | 1672 (22.45) | 1976 (26.78) | 74 (25.70) | 390 (27.62) | <0.001 |
| Low to moderate drinker | 6312 (42.27) | 1493 (43.77) | 2261 (43.89) | 2103 (40.94) | 67 (35.01) | 388 (36.47) |  |
| Heavy drinker | 5274 (33.84) | 1155 (35.54) | 1885 (33.66) | 1779 (32.28) | 85 (39.29) | 370 (35.91) |  |
| **Body mass index (mean ± SD ^b^)** |  |  |  |  |  |  |  |
| At age 25 years | 23.73 ± 0.06 | 20.57 ± 0.04 | 22.87 ± 0.05 | 24.20 ± 0.06 | 34.36 ± 0.43 | 34.69 ± 0.18 | <0.001 |
| At survey | 29.64 ± 0.09 | 22.32 ± 0.04 | 27.25 ± 0.03 | 35.06 ± 0.08 | 26.96 ± 0.18 | 40.06 ± 0.33 | <0.001 |
| **Absolute weight change (mean ± SD ^b^, kg)** | 15.49 ± 0.19 | 4.10 ± 0.16 | 11.37 ± 0.16 | 28.94 ± 0.24 | -20.62 ± 1.37 | 13.81 ± 1.01 | <0.001 |

^a^ All estimates accounted for complex survey designs. Data are expressed as No. (%) unless otherwise indicated.

^b^ SD, Standard Deviation.

**Table S3.** **Odd ratios (OR) and 95% confidence intervals (CIs) of depression with BMI status in the NHANES 2005-2018. ^a^**

| **Weight status ^b^** | **No. of depression / No. of participants** | **Model 1 ^c^** | | **Model 2 ^d^** | | **Model 3 ^e^** | |
| --- | --- | --- | --- | --- | --- | --- | --- |
|  |  | **OR (95% CI)** | ***P*** | **OR (95% CI)** | ***P*** | **OR (95% CI)** | ***P*** |
| **BMI at age 25 years** | | | | | | | |
| Underweight | 121/1198 | 1.49 (1.13, 1.97) | 0.006 | 1.32 (1.00, 1.75) | 0.054 | 1.15 (0.86, 1.55) | 0.345 |
| Normal (ref) | 957/10 913 | 1.00 |  | 1.00 |  | 1.00 |  |
| Overweight | 403/3991 | 1.10 (0.94, 1.28) | 0.252 | 1.30 (1.10, 1.55) | 0.003 | 1.33 (1.12, 1.57) | 0.001 |
| Obesity | 191/1454 | 1.50 (1.21, 1.85) | <0.001 | 1.57 (1.27, 1.94) | <0.001 | 1.43 (1.14, 1.78) | 0.002 |
| **BMI at 10 years before survey** | | | | | | | |
| Underweight | 28/262 | 1.10 (0.71, 1.71) | 0.675 | 0.96 (0.62, 1.51) | 0.874 | 0.68 (0.43, 1.07) | 0.095 |
| Normal (ref) | 485/5846 | 1.00 |  | 1.00 |  | 1.00 |  |
| Overweight | 545/6261 | 1.03 (0.89, 1.20) | 0.671 | 1.21 (1.04, 1.40) | 0.016 | 1.23 (1.05, 1.44) | 0.012 |
| Obesity | 614/5187 | 1.42 (1.19, 1.70) | <0.001 | 1.61 (1.35, 1.92) | <0.001 | 1.62 (1.35, 1.93) | <0.001 |
| **BMI at survey** | | | | | | | |
| Underweight | 26/199 | 2.02 (0.92, 4.44) | 0.079 | 1.95 (0.90, 4.26) | 0.092 | 1.26 (0.50, 3.18) | 0.615 |
| Normal (ref) | 310/3990 | 1.00 |  | 1.00 |  | 1.00 |  |
| Overweight | 455/5980 | 0.97 (0.78, 1.21) | 0.809 | 1.08 (0.86, 1.34) | 0.507 | 1.13 (0.90, 1.40) | 0.286 |
| Obesity | 881/7387 | 1.54 (1.26, 1.88) | <0.001 | 1.63 (1.33, 1.99) | <0.001 | 1.69 (1.39, 2.07) | <0.001 |

^a^ All estimates accounted for complex survey designs.

^b^ We categorized BMI variables into four groups: underweight (<18.5) and normal weight (18.5-24.9), overweight (25.0-29.9), and obesity (≥30).

^c^ Model 1 was unadjusted.

^d^ Model 2 was adjusted for age and sex.

^e^ Model 3 was additionally adjusted for race/ethnicity, education level, family income-poverty ratio level, smoking status, and drinking status.
